# A new species of cryptic Bush frog (Anura, Rhacophoridae, *Raorchestes*) from northeastern Bangladesh

**DOI:** 10.3897/zookeys.927.48733

**Published:** 2020-04-16

**Authors:** Hassan Al-Razi, Marjan Maria, Sabir Bin Muzaffar

**Affiliations:** 1 Faculty of Life and Earth Science, Department of Zoology, Jagannath University, Dhaka, Bangladesh Jagannath University Dhaka Bangladesh; 2 Department of Biology, United Arab Emirates University, Al Ain, United Arab Emirates Jagannath University Al Ain United Arab Emirates

**Keywords:** Amphibian, bush frog, DNA, herpetofauna, *Raorchestes rezakhani* sp. nov.

## Abstract

*Raorchestes* is a speciose genus of bush frogs with high diversity occurring in the Western Ghats of India. Relatively fewer species have been recorded across India, through Bangladesh, southern China, into Vietnam and Peninsular Malaysia. Many bush frogs are morphologically cryptic and therefore remain undescribed. Here, a new species, *Raorchestes rezakhani***sp. nov.**, is described from northeastern Bangladesh based on morphological characters, genetics, and bioacoustics. The 16S rRNA gene distinguished this species from 48 known species of this genus. Bayesian Inference and Maximum Likelihood analyses indicated that the new species was most similar to *R. tuberohumerus*, a species found in the Western Ghats, and to *R. gryllus*, a species found in Vietnam. Bioacoustics indicated that their calls were similar in pattern to most *Raorchestes* species, although number of pulses, duration of pulses, pulse intervals and amplitude differentiated it from a few other species. It is suggested that northeastern India, Bangladesh, northern Myanmar, and southern China represent important, relatively unexplored areas that could yield additional species of *Raorchestes*. Since many remaining habitat patches in Bangladesh are under severe threat from deforestation, efforts should be made to protect these last patches from further degradation.

## Introduction

*Raorchestes*Biju et al., 2010, is a genus of bush frogs belonging to the family Rhacophoridae, that extends in distribution from southwestern India through northeastern India, Bangladesh, Myanmar, southern China, and into Laos, Vietnam, and Peninsular Malaysia (Biju and Bossuyt 2009; IUCN 2016; Vijayakumar et al. 2016; Frost 2019). The genus is particularly speciose in the Western Ghats of India, where more than 50 of the 63 recorded species occur (Biju et al. 2010; Vijayakumar et al. 2014; Priti et al. 2016; Vijayakumar et al. 2016; Boruah et al. 2018). In addition, a few species have been recorded from the Eastern Ghats, Eastern Himalayas, and northeastern India, southern China and adjoining regions (Vijayakumar et al. 2016; Boruah et al. 2018; Wu et al. 2019, Frost 2020). Many species in the genus are cryptic (morphologically difficult to distinguish from congenerics) and, as a result, remain undescribed (Priti et al. 2016; Vijayakumar et al. 2016; Boruah et al. 2018; Wu et al. 2019). The genus is characterized within the Rhacophoridae by small size (15–45 mm snout-vent length), absence of vomerine teeth, transparent gular pouch, and direct development (Biju et al. 2010). Their advertisement calls consists of repetitive ‘treenk.. treenk.. treenk’ with variation in number of pulses, duration of calls, interval between calls and amplitude, which may be used to distinguish between species (Priti et al. 2016). Thus, an integrative approach using morphological traits, bioacoustics and molecular variation has been used to distinguish cryptic species (Priti et al. 2016; Boruah et al. 2018).

Bangladesh falls within the Indo-Malayan realm, with forests classified as tropical moist, tropical evergreen and several other less-extensive forest types (Champion and Seth 1968; Slik et al. 2018). Broad similarities exist between forest patches in northeastern and southeastern Bangladesh and the surrounding Indian States of Meghalaya, Tripura, Mizoram and Nagaland, and adjoining northern Myanmar and southern China (Slik et al. 2018). Three species of *Raorchestes*, namely the Darjeeling bush frog *Raorchestes annandalii* (Boulenger, 1906), the Karin bubble-nest frog *R. parvulus* (Boulenger, 1893) and, most recently, the Longchuan bush frog *R. longchuanensis* (Yan and Li 1978) have been recorded from Bangladesh (Ghose and Bhuiyan 2012; IUCN 2015; Khan 2015; Al Razi et al. 2020). *Raorchestes parvulus* has a distribution from northeastern India, Bangladesh through Southeast Asia extending up to Vietnam and Peninsular Malaysia (Khan 2015; IUCN 2015; IUCN-SSC 2016). In fact, *R. parvulus* has been confused with *R. longchuanensis* reported originally from southern China (Frost 2020) and recently from northeastern Bangladesh (Al-Razi et al. 2020). Furthermore, Thai populations of *R. parvulus* are possibly separate species (Frost 2020). Thus, *R. parvulus* has been regarded as members of a species complex (Khan 2015; IUCN 2015; IUCN-SSC 2016). It has been speculated that *R. longchuanensis* has a wider distribution in northeastern India and northern Myanmar (Al-Razi et al. 2020). *Raorchestes annandalii*, on the other hand, has a more restricted distribution in southeastern Bangladesh, northeastern India, and Nepal (Bardoloi et al. 2004; IUCN 2015). The region of northeastern India that surrounds Bangladesh hosts at least four species of *Raorchestes* and other related genera. This suggests that the region represent a zone of diversification of bush frogs. It is possible that *R. annandalii* represents a northern complex of related species and *R. parvulus* are part of a more southern species complex (Frost 2020). Here we describe a new species of bush frog from northeastern Bangladesh based on bioacoustics, morphology, and molecular characterization.

## Materials and methods

### Study area

**Figure 1.**
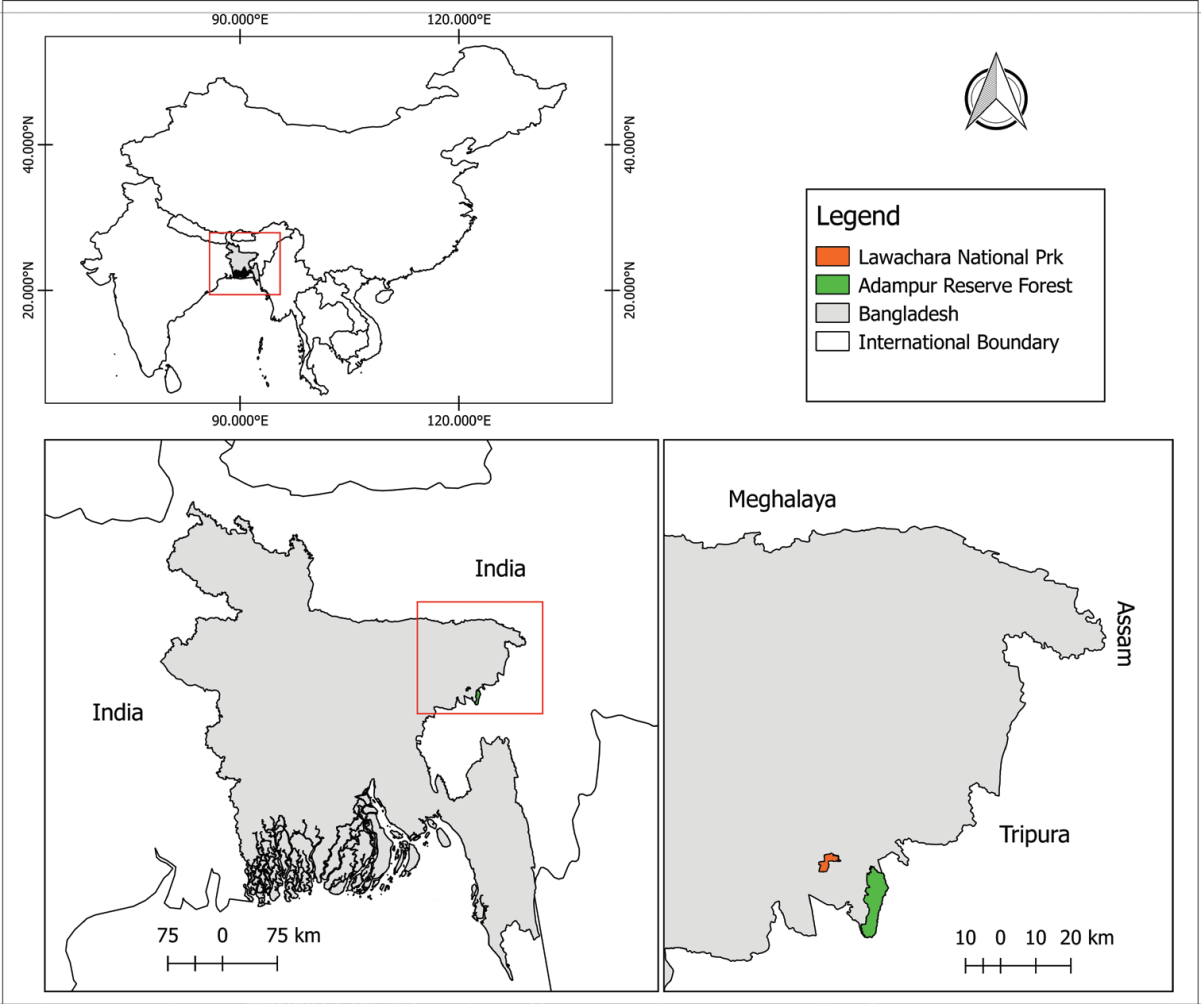
Map showing the type location of *Raorchestes rezakhani* sp. nov. in northeastern Bangladesh as well as adjoining areas.

We conducted this study in Adampur Reserve Forest (24°13.410'N, 91°54.836'E) and Lawachara National Park (24.330755N, 91.789396E), two small forest patches of northeastern Bangladesh (Fig. 1). Both forests are semi-evergreen, and local climate and hydrologic patterns are similar, but their sizes and disturbance patterns differ (Quazi and Ticktin 2016). The topography of the study area is hilly, with elevations ranging from 50–100 m a.s.l. (Islam et al. 2007). Annual temperature ranges from 9 °C (January) to 32 °C (August-October), and nearly 80% of the annual average rainfall (3,334 mm) occurs between the months of May and October (Quazi and Ticktin 2016). Numerous streams and swampy areas crisscross the region. The landscape is categorized into hill forests, scrublands, and mixed bamboo forests (IUCN 2015). Northeast Bangladesh shares an international border with India, and two of the Indian states, Tripura, and Assam, are adjacent to northeast Bangladesh (Fig. 1).

### Specimen collection

We collected four adult calling males from April to October 2019. We euthanized and fixed the specimens in 95% ethanol for 5 hrs and stored them in 70% ethanol. We tentatively designated the specimens to the genus *Raorchestes* based on small size (18.85–20.90 mm snout-vent length), absence of vomerine teeth, transparent gular pouch, and advertisement calls consisting of repetitive ‘treenk.. treenk.. treenk’ which is characteristic of *Raorchestes* (following Biju et al. 2010). Thigh-muscle samples for genetic analysis were collected before fixing the specimen. We recorded the color of living specimens and recorded natural history observations at the type locality during specimen collections. As the frog was very small, cryptic, and very difficult to find, we were able to collect only four specimens. We deposited the specimens in the Shahid Rafique Special Specimen Collection (**SRSSC**), Department of Zoology, Jagannath University, Dhaka. Since the SRSSC is a newly established part of the Zoological Museum, the catalogue numbers for specimens retain the original codes adopted by the Zoological Museum, namely JnUZool.

### Morphometrics

We measured the following from the left side of the specimens with digital calipers (to the nearest 0.10 mm):

**ED** eye diameter (horizontal diameter of the eye);

**EN** eye-nostril distance (distance between anterior canthus of eye and the posterior edge of nostril);

**FD I to IV** width of 1^st^ to 4^th^ finger disks (measured at the widest point on the finger disk);

**FL I to** lengths of 1^st^ to 4^th^ fingers (from the tip of the respective finger to where it;

**FL IV** connects with the palm);

**FOL** foot length (from the distal end of tarsus tip of Toe IV);

**HAL** hand length (from distal end of radioulna to tip of distal finger III);

**HL** head length (distance between tip of the snout to the rear of the mandible);

**HW** head width (at angle of jaw);

**IND** internarial distance (least distance between inner edge of the nostrils);

**IOD** interorbital distance (least distance between proximal edges of upper eyelids);

**NS** nostril-Snout distance (distance from the anterior edge of nostril to the tip of the snout);

**ShL** shank length (distance between knee and heel);

**SL** snout length (from anterior canthus of eye to tip of snout);

**SVL** snout-vent length (from tip of snout to vent);

**TD** tympanum diameter (maximum diameter of the tympanum);

**TD I to V** width of 1^st^ to 5^th^ toe disks (the greatest horizontal distance between the edges of toe disks);

**TL** thigh length (distance from the middle of vent to knee);

**TL I to V** lengths of 1^st^ to 5^th^ toes (from base of proximal subarticular tubercle to tip of the respective toe);

**UEW** upper eyelid width (maximum transverse distance of the upper eyelid.

We compared morphological characters based on morphometric measurements provided in the following published papers (Al-Razi et al. 2020; Kuramoto and Joshy 2003; Padhye et al. 2015; Orlov et al. 2012):

In addition, we compared eleven linear, morphometric variables of four species using Principle Components Analysis (PCA) (McGarigal et al. 2000; Sokal and Rohlf 2012), using PAST (version 3.8). All linear morphometric variables were transformed by subtracting each variable from the mean of that variable (McGarigal et al. 2000; Sokal and Rohlf 2012). We derived eleven principle components, since there were eleven variables, each representing a linear combination of all eleven variables. We generated Eigenvalues and their relative weightings to determine the relative contribution of the variables towards each principle component (McGarigal et al. 2000). Loadings of each of the eleven variables in relation to each of the eleven principle components were used to determine relative effect of individual morphological characters on each principle component. We visualized the differences in the species compared using a scatter plot of principle components that explained the greatest variance in the data (McGarigal et al. 2000).

### DNA Extraction and amplification

We extracted DNA from the muscle samples using a standard protocol described in Vences et al. (2012) for DNA extraction. We amplified mitochondrial 16S ribosomal RNA gene. The PCR amplification and sequencing of the 16S rRNA gene were done following Palumbi et al. (1991) and Bossuyt et al. (2004) respectively. We used primers 5’ -GCCTGTTTATCAAAAACAT-3’ (16Sar-L) and 5’ -CCGGTCTGAACTCAGATCACGT-3’ (16Sbr-H) as forward and reverse primers for 16S (Palumbi et al. 1991) for this study. We performed PCR amplifications in a 20 μl reaction volume; Master Mix 10 μl, T DNA (Concentration 25–65 ng/ μl) 1 μl, Primer F (Concentration 10–20 pMol) 1 μl, Primer R (Concentration 10–20 pMol) 1 μl and nuclease-free water 7 μl with the following cycling conditions: an initial denaturing step at 95 °C for 3 min; 40 cycles of denaturing at 95 °C for 30 s, annealing at 50 °C for 30 s and extending at 72 °C for 45 s, and a final extension step of 72 °C for 5 min. We sent the amplified product to First Base Laboratories, Malaysia for sequencing. The sequences were checked manually using the program Chromas lite 2.01 (http://www.technelysium.com.au/chromas_lite.html). The sequences were submitted to GenBank (Accession no: MN072374, MN072375, MN615901, MN615902).

### Phylogenetic analyses

We compared the new sequences to the GenBank sequences using the BLAST tool (http://blast.ncbi.nlm.nih.gov/Blast.cgi) in order to confirm their genetic identity and determine similar species that allow the evaluation of the phylogenetic position of the new taxon. Homologous sequences of other *Raorchestes* species were obtained from GenBank (Table 1). *Kurixalus eiffingeri* Boettger, 1895 was selected as outgroup based on Yu et al. (2013). Sequences were aligned using the MUSCLE tool in MEGA 7 (Kumar et al. 2016), alignments were checked visually, and both ends of the sequence were trimmed to avoid low quality base pairs. Alignment gaps were treated as missing data. The best substitution model (GTR+I+G) was selected using the Akaike Information Criterion (AIC) and Bayesian information criteria (BIC) in jModelTest v2.1.2. Maximum likelihood phylogenetic analyses were performed using the RAxML v4.0 Geneious plugin (Stamatakis 2006) with 1,000 bootstrap replicates. Bayesian phylogenetic inference analysis were performed in MrBayes 3.2.4 (Ronquist et al. 2012). We performed an MCMC Bayesian analysis that consisted of two simultaneous runs of 1 million generations and sampled every 100 generations. The first 25% of the sampled trees were discarded as burn-in, and the remaining trees were used to create a consensus tree and to estimate Bayesian posterior probabilities (BPPs). The trees were visualized and edited in FigTree 1.4.4 (http://tree.bio.ed.ac.uk/software/figtree). Additionally, pairwise genetic distances (uncorrected *p*) of 21 species under the genus *Raorchestes* including the new species were calculated for 16S using MEGA 7.0 (Kumar et al. 2016).

**Table 1.** Species of *Raorchestes* and the outgroup and their associated GenBank accession numbers that were used in the phylogenetic analysis.

	Species	Location	Voucher	GenBank16S rRNA accession numbers	Source
1	*R. rezakhani* sp. nov.	Maulovibazar, Bangladesh	JnUZool-A0319	MN072374	This study
2	*R. rezakhani* sp. nov.	Maulovibazar, Bangladesh	JnUZool-A0419	MN072375	This study
3	*R. rezakhani* sp. nov.	Maulovibazar, Bangladesh	JnUZool-A0619	MN615901	This study
4	*R. rezakhani* sp. nov.	Maulovibazar, Bangladesh	JnUZool-A0519	MN615902	This study
5	*R. longchuanensis*	Habigonj, Bangladesh	JnUZool-A0317	MN193414	Al-Razi et al. 2020
6	*R. longchuanensis*	Habigonj, Bangladesh	JnUZool-A0117	MN193412	Al-Razi et al. 2020
7	*R. ghatei*	Satara, Maharashtra, India	WILD-AMP-13-100	KF366385	Padhye et al. 2013
8	*R. ghatei*	Satara, Maharashtra, India	ZSI-WRC A/1484	KF366384	Padhye et al. 2013
9	*R. ghatei*	Satara, Maharashtra, India	WILD-AMP-13-104	KF366387	Padhye et al. 2013
10	*Raorchestes* sp. R3	Riwai, Meghalaya,India	–	MG980284	Boruah et al. 2018
11	*Raorchestes* sp. R4	Mawlynong, Meghalaya,India	–	MG980285	Boruah et al. 2018
12	*R. shillongensis*	Malki forest, Meghalaya,India	–	MG980282	Boruah et al. 2018
13	*R. shillongensis*	Malki forest, Meghalaya,India	–	MG980283	Boruah et al. 2018
14	*R. gryllus*	Pac Ban, Vietnam	ROM30288	GQ285674	Li et al. 2009
15	*R. menglaensis*	Yunnan, China	KIZ060821286	EU924621	Yu et al. 2009
16	*R. bombayensis*	Uttara Kannada, Karnataka, India	1362PhiBom	EU450019	Biju and Bossuyt 2009
17	*R. bombayensis*	Uttara Kannada, Karnataka, India	WILD-13-AMP-230	KF767502	Padhye et al. 2013
18	*R. tuberohumerus*	Western Ghats, India	CESF424 16S	KM596574	Vijayakumar et al. 2009
19	*R. tuberohumerus*	Western Ghats, India	0073PhiTub	EU450004	Biju and Bossuyt 2009
20	*R. sanctisilvaticus*	Eastern Ghats, India	SKD244	MH915511	Mirza et al. 2019
21	*R. sanctisilvaticus*	Eastern Ghats, India	SKD240	MH915509	Mirza et al. 2019
22	*R. ponmudi*	Western Ghats, India	1451PhiPonb	EU450026	Biju and Bossuyt 2009
23	*R. ponmudi*	Western Ghats, India	1121PhiPon	EU450011	Biju and Bossuyt 2009
24	*R. ponmudi*	Western Ghats, India	0030PhiBed	EU449998	Biju and Bossuyt 2009
25	*R. indigo*	Western Ghats, India	CESF138	KM596557	Vijayakumar et al. 2009
26	*R. parvulus*	southern Yunnan, China	KIZ 20160374	MK564634	Yu et al. 2019
27	*R. parvulus*	southern Yunnan, China	KIZ 20160366	MK564630	Yu et al. 2019
28	*R. theuerkaufi*	Western Ghats, India	CESF1342	JX092693	Vijayakumar et al. 2009
29	*R. signatus*	Western Ghats, India	CESF1666	KM596562	Vijayakumar et al. 2009
30	*R. signatus*	Western Ghats, India	CESF1662	KM596561	Vijayakumar et al. 2009
31	*R. tinniens*	Munnar, Kerala, India	SDBDU2010.274	KU169991	Biju et al. 2016
32	*R. tinniens*	Western Ghats, India	0058PhiTin	EU450001	Biju and Bossuyt 2009
33	*R. marki*	Western Ghats, India	CESF467	JX092719	Vijayakumar et al. 2009
34	*R. chromasynchysi*	Western Ghats, India	CESF1127	JX092667	Vijayakumar et al. 2009
35	*R. chromasynchysi*	Western Ghats, India	CESF1203	KM596543	Vijayakumar et al. 2009
36	*R. charius*	Karnataka, India	SDBDU2011.814	KU169985	Biju et al. 2016
37	*R. charius*	Sri Lanka	–	AY141840	Meegaskumbura et al. 2002
38	*R. primarrumfi*	Western Ghats, India	CESF442	KM596575	Vijayakumar et al. 2009
39	*R. chalazodes*	Western Ghats, India	BRA-2014	KJ619643	Unpublished
40	*R.* sp.	Western Ghats, India	CESF403	JX092710	Vijayakumar et al. 2009
41	*R.* sp.	Western Ghats, India	CESF427	JX092714	Vijayakumar et al. 2009
42	*R.* sp.	Western Ghats, India	SPV-2014b	KM596563	Vijayakumar et al. 2009
43	*R. lechiya*	Western Ghats, India	CB-2015a	KT359622	Zachariah et al. 2016
44	*R. lechiya*	Western Ghats, India	CB-2015a	KT359623	Zachariah et al. 2016
45	*R.* sp.	Western Ghats, India	SPV-2014b	KM596563	Vijayakumar et al. 2009
46	*Kurixalus eiffingeri*	Okinawa Islands, Japan	A120	DQ468673	Wu et al. 2016

### Call recording and analysis

The call of a single male individual (JnUZool- A0519) was recorded with a Sony ICD-PX240 digital sound recorder with sampling rate of 48 kHz and 32-bit resolution on 10 May 2019. The device was approximately 1–1.5 m away from the calling male. Air temperature and humidity were taken by a digital hygrometer. For the call analysis we used Raven Pro Ver. 1.5 (Charif et al. 2010; Bioacoustics Research Program 2011). We measured call-group duration, inter-call group interval, duration of intervals between pulses, call duration, pulse rate and dominant frequency comprising of 25 call groups.

## Results

### Molecular data

**Figure 2.**
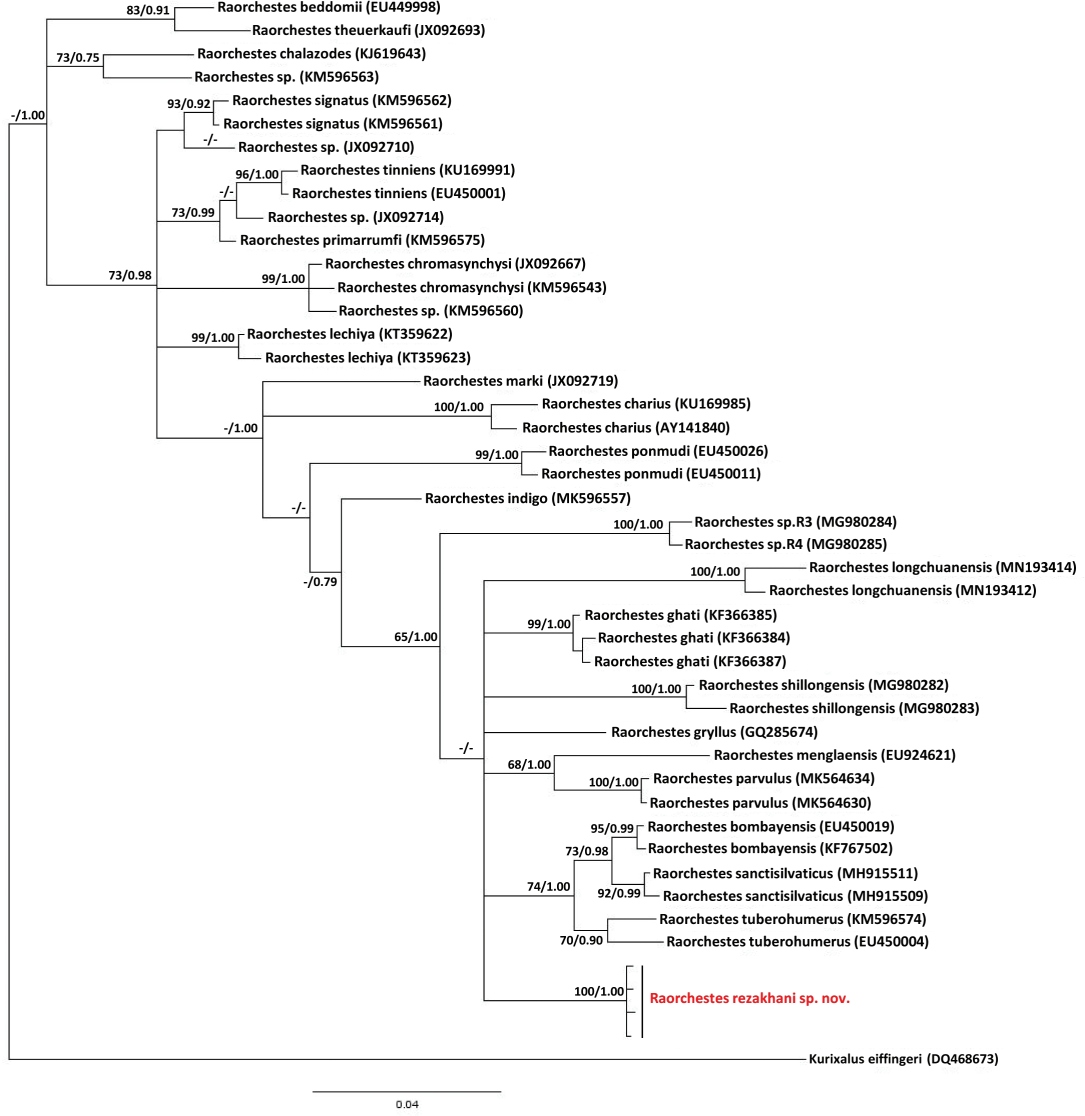
Bayesian Inference and Maximum Likelihood phylogenies, showing the placement of *Raorchestes rezakhani* sp. nov. in relation to other congeneric species. The Bayesian Posterior Probabilities (BPP) > 0.75 and the bootstrap supports for Maximum Likelihood analysis (ML) > 60 were retained.

The ML and BI analyses resulted in essentially identical topologies and were integrated in the consensus tree (Fig. 2), in which the maximum nodes were sufficiently supported with the Bayesian posterior probabilities (BPP) > 0.90 and the bootstrap supports (BS) for maximum likelihood analysis > 70 and a few poorly supported basal nodes. Both Bayesian and Maximum Likelihood analyses strongly supported that the new species is in the genus *Raorchestes*. The uncorrected p-distances for the 16S rRNA gene that are interpreted as interspecific distances were lowest between *R. bombayensis* Annandale 1919 and *R. sanctisilvaticus* Das and Chanda 1997 (*p* = 1.4%, Table 2). The highest interspecific distances were between *R. tinniens* (Jerdon 1854) and *R. ghatei*Padhye et al. 2013 (*p* = 10.6%, Table 2). The newly discovered species was most similar to *R. tuberohumerus* (Kuramoto and Joshy 2003) (*p* = 4.6%) followed by *R. gryllus* (Smith 1924) (*p* = 4.7%) at the gene fragment examined. In addition, *R. rezakhani* sp. nov. differed considerably from *R. longchuanensis* (*p* = 5.5%), *R. shillongensis* (*p* = 5.5%) and R. parvulus (*p* = 6.6%, Table 2). The average divergence (p-distance) between the new species and other congeneric species ranged from 4.6% to 9.6% (Table 2). This level of divergence in the 16S rRNA gene is typically seen in many other frog species pairs, thereby justifying the status of *R. rezakhani* sp. nov. as a new species (Fouquet et al. 2007).

**Figure 3.**
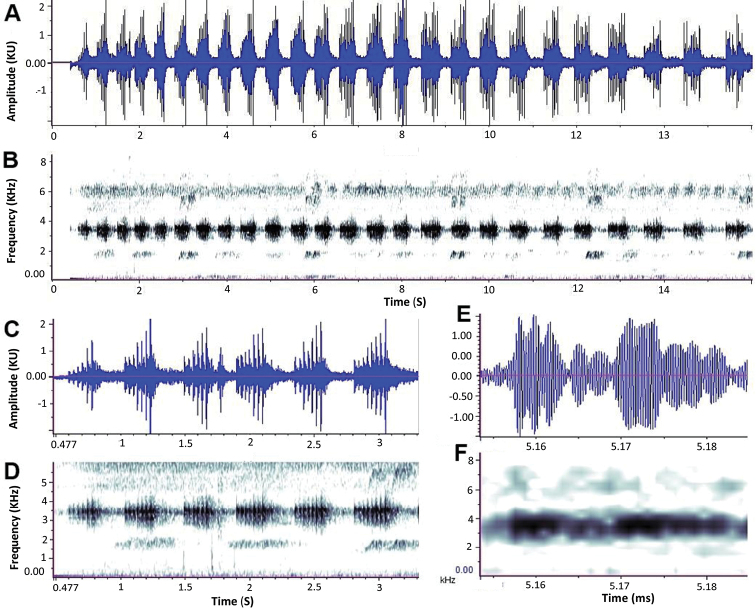
Advertisement call of *Raorchestes rezakhani* sp. nov. showing 25 notes that vary in amplitude **A** waveform of 25 notes **B** shows variation in frequency **C** shows waveform of first six notes of the call; and **D** shows a spectrogram of the six notes **E** shows a pulse of fourth note and **F** shows the spectrogram of pulse of fourth note.

**Table 2.** Intraspecific and interspecific genetic divergence. Uncorrected p-distances between 16S rRNA sequences of closely related 20 species of *Raorchestes*.

	Species name	1	2	3	4	5	6	7	8	9	10	11	12	13	14	15	16	17	18	19	20	21
**1**	*R. rezakhani* sp. nov.	–																				
**2**	*R. longchuanensis*	0.055	–																			
**3**	*R. shillongensis*	0.055	0.072	–																		
**4**	*R. ghatei*	0.065	0.085	0.061	–																	
**5**	*R. gryllus*	0.047	0.067	0.054	0.058	–																
**6**	*R. menglaensis*	0.059	0.066	0.054	0.065	0.065	–															
**7**	*R. bombayensis*	0.061	0.074	0.044	0.071	0.056	0.063	–														
**8**	*R. tuberohumerus*	0.046	0.058	0.037	0.063	0.053	0.051	0.019	–													
**9**	*R. sanctisilvaticus*	0.061	0.067	0.047	0.070	0.058	0.062	0.014	0.014	–												
**10**	*R. ponmudi*	0.074	0.077	0.067	0.096	0.087	0.086	0.084	0.076	0.084												
**11**	*R. beddomii*	0.072	0.082	0.07	0.095	0.084	0.087	0.081	0.07	0.082	0.061	–										
**12**	*R. indigo*	0.054	0.063	0.039	0.069	0.059	0.058	0.055	0.053	0.054	0.054	0.064	–									
**13**	*R. parvulus*	0.066	0.065	0.06	0.072	0.058	0.047	0.044	0.045	0.042	0.083	0.088	0.061	–								
**14**	*R. theuerkaufi*	0.078	0.088	0.078	0.093	0.088	0.088	0.077	0.064	0.075	0.067	0.021	0.062	0.082	–							
**15**	*R. signatus*	0.082	0.097	0.073	0.088	0.087	0.076	0.078	0.074	0.078	0.072	0.060	0.043	0.081	0.063	–						
**16**	*R. tinniens*	0.084	0.105	0.075	0.106	0.099	0.088	0.078	0.072	0.08	0.082	0.071	0.048	0.097	0.077	0.028	–					
**17**	*R. marki*	0.069	0.069	0.067	0.077	0.071	0.066	0.067	0.061	0.062	0.064	0.066	0.047	0.059	0.064	0.051	0.056	–				
**18**	*R. chromasynchysi*	0.082	0.084	0.064	0.078	0.085	0.068	0.08	0.074	0.083	0.073	0.060	0.055	0.085	0.064	0.044	0.050	0.051	–			
**19**	*R. charius*	0.096	0.105	0.071	0.096	0.091	0.08	0.084	0.070	0.087	0.085	0.063	0.064	0.091	0.073	0.078	0.080	0.058	0.075	–		
**20**	*R. primarrumfi*	0.073	0.085	0.059	0.084	0.073	0.076	0.063	0.070	0.066	0.058	0.049	0.046	0.077	0.054	0.021	0.012	0.054	0.038	0.058	–	
**21**	*R. chalazodes*	0.081	0.091	0.085	0.085	0.085	0.079	0.079	0.076	0.079	0.087	0.058	0.065	0.085	0.058	0.044	0.057	0.057	0.052	0.077	0.049	–

#### Raorchestes rezakhani sp. nov.

Taxon classificationAnimaliaAnuraRhacophoridae

3CD826E6-7748-51DB-9F11-7E7D4C16CE6C

http://zoobank.org/CDDA555B-9B29-4D94-B5E6-BA560ECD8DB3

**Figure 4.**
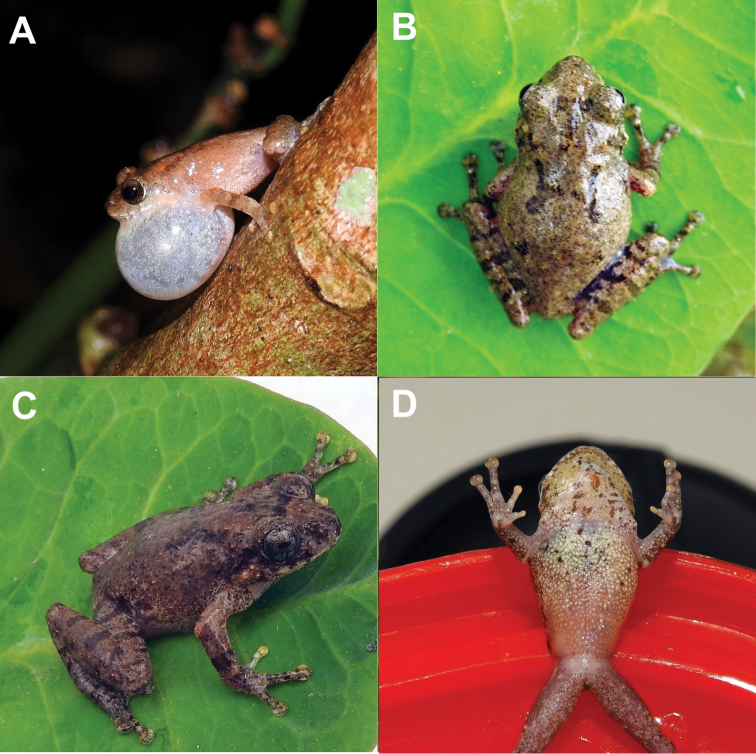
Color variation in *R. rezakhani* sp. nov. **A** holotype, showing single transparent vocal sac during advertisement call (**B** holotype with brown dorsum and “)-(“ mark; **C** dorsolateral view of paratype (JnUZool- A0519) **D** ventral view of paratype (JnUZool- A0519), showing small dark brown spots.

**Figure 5.**
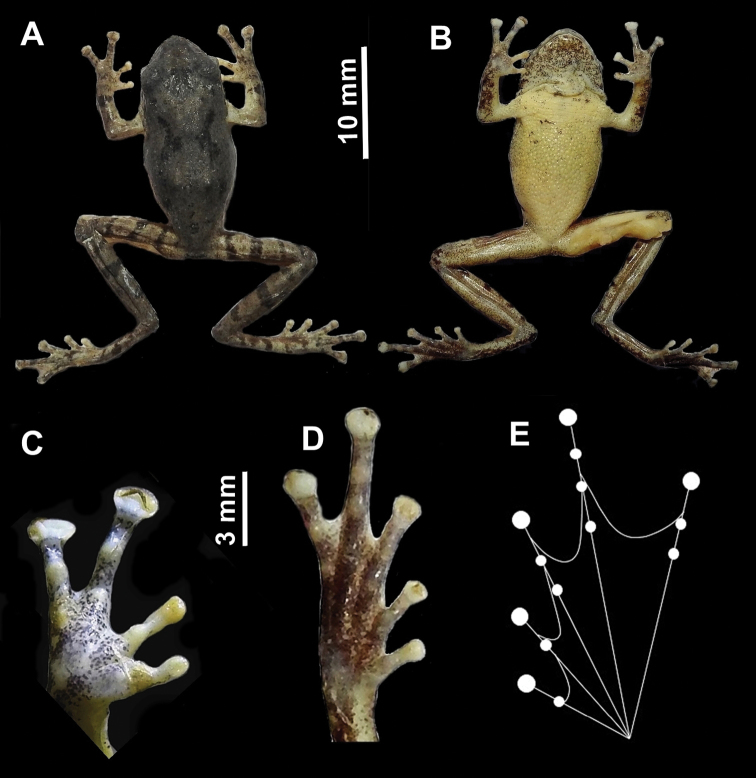
Holotype of *R. rezakhani* sp. nov. **A** dorsal view **B** ventral view **C** ventral view of right hand **D** ventral view of right foot **E** web pattern in foot.

Figures 4 , 5 Suggested English name: Reza Khan’s bush frog

##### Type.

***Holotype*** (Figs 4A, B, 5). JnUZool-A0419, an adult male from Lawachara National Park, Kamalgonj, Moulavibazar, Bangladesh (24°20.746'N, 91°47.945'E, ca. 59 m a.s.l., Fig. 1), collected on 26 April 2019 by Hassan Al-Razi and Marjan Maria.

***Paratypes*** (Fig. 4C, D). Three specimens: adult male (JnUZool-A0319) same locality as the holotype; two adult males (JnUZool-A0519, JnUZool-A0619) from the Adampur, Rajkandhi Reserved Forest, Kamalgonj, Moulavibazar (24°14.878'N, 91°54.002'E, ca. 64 m a.s.l., Fig. 1), on 10 May 2019 by Hassan Al-Razi.

##### Generic placement.

We assign this species to *Raorchestes* based on molecular characterization of the 16S rRNA gene.

##### Etymology.

We take great pleasure in naming the new species as a patronym for one of the pioneers in the field of wildlife research in Bangladesh, Dr. Mohammad Ali Reza Khan.

##### Diagnosis.

A species of *Raorchestes* having the following unique combination of characters: (1) relatively small size (adult males = 18.85–20.90 mm SVL); (2) head wider than long (HW/HL 1.55; range 1.53–1.56, *N* = 4); (3) dark brown, granular dorsum bearing small, horny spicules; (4) vomerine teeth absent; (5) single transparent vocal sac while calling; (6) snout projecting, sub-elliptical in ventral aspect, and subequal to or smaller than horizontal diameter of eye; (7) tympanum indistinct; (8) supratympanic fold weakly distinct; (9) finger and toe discs well developed and rounded; (FD IV 0.50–0.60, TD IV 0.56–0.65 mm); (10) both inner and outer metacarpal and metatarsal tubercles absent; (11) nostril is closer to tip of snout than to eye (NS 0.63–0.90, EN 1.10–1.25 mm); (12) Tongue without papilla (13) venter pale white, with minute dark gray flecks present in the vocal sac region. Details of these measurements are provided in Table 3.

##### Description of holotype.

A small frog (SVL = 20.30, Fig. 5, Table 3, all measurements in mm); head wider than long (HW = 7.0; HL = 4.5); snout sub-elliptical in ventral aspect, shorter than eye diameter (ED = 2.45; SL = 2.24). Canthus rostralis sharply rounded; loreal region slightly concave. Interorbital region flat and larger (IOD = 2.20) than the upper eyelid (UEW = 1.45 mm) or internarial distance (IND = 1.70). Nostrils oval (dorsally compressed), without flap, directed laterally, closer to tip of snout than to eye (NS = 0.80; EN = 1.20). Tympanum indistinct, oval (TD = 1.10), close to eye, supratympanic fold weakly distinct, extends from eye to the end of the tympanum. Vocal sac single, sub-gular, translucent. Tongue bifid, lingual papilla absent. Eyes relatively large (ED = 2.45), protruding; pupil horizontal.

**Table 3.** Morphological measurements (in mm) of the four specimens of *Raorchestes rezakhani* sp. nov.

	**Characters**	**Abbreviation**	**Holotype**	**Paratype**			**Mean** ± **SD**
			**JnUZool-A0419**	**JnUZool-A0319**	**JnUZool- A0519**	**JnUZool-A0619**	
1	Snout–vent length	SVL	20.30	20.90	20.20	18.85	20.06 ± 0.87
2	Head length	HL	4.50	4.60	4.55	4.50	4.54 ± 0.05
3	Head width	HW	7.00	7.05	7.10	6.95	7.03 ± 0.06
4	Eye diameter	ED	2.45	2.70	2.50	2.65	2.58 ± 0.12
5	Tympanum diameter	TD	1.10	1.22	1.21	1.16	1.17 ± 0.05
6	Eye–nostril distance	EN	1.25	1.20	1.10	1.20	1.19 ± 0.06
7	Snout length	SL	2.24	2.25	2.24	2.22	2.24 ± 0.01
8	Nostril-Snout distance	NS	0.80	0.90	0.85	0.63	0.80 ± 0.12
9	Interorbital distance	IOD	2.20	2.40	2.25	2.20	2.26 ± 0.09
10	Internarial distance	IND	1.70	1.65	1.60	1.70	1.66 ± 0.05
11	Upper eyelid width	UEW	1.45	1.50	1.40	1.55	1.48 ± 0.06
12	Thigh length	TL	10.03	9.20	10.10	10.00	9.83 ± 0.42
13	Shank length	ShL	10.10	10.10	11.90	10.20	10.58 ± 0.88
14	Foot length	FOL	7.95	6.60	7.85	7.95	7.59 ± 0.66
15	Hand length	HAL	4.90	4.35	4.95	4.90	4.78 ± 0.28
16	Fore limb length	FLL	4.70	4.70	5.0	5.0	4.85 ± 0.17
17	Finger I disk width	FD I	0.25	0.20	0.20	0.20	0.21 ± 0.02
18	Finger II disk width	FD II	0.45	0.40	0.40	0.40	0.41 ± 0.03
19	Finger III disk width	FD III	0.75	0.70	0.75	0.70	0.73 ± 0.03
20	Finger IV disk width	FD IV	0.50	0.50	0.60	0.50	0.53 ± 0.05
21	Finger I length	FL I	1.20	1.05	1.10	1.20	1.14 ± 0.07
22	Finger II length	FL II	1.75	1.80	1.70	1.80	1.76 ± 0.05
23	Finger III length	FL III	3.40	3.05	3.55	3.55	3.39 ± 0.24
24	Finger IV length	FL IV	2.15	1.95	2.20	2.25	2.14 ± 0.13
25	Toe I length	TL I	1.15	1.00	1.00	1.15	1.08 ± 0.09
26	Toe II length	TL II	2.10	1.90	2.05	1.90	1.99 ± 0.10
27	Toe III length	TL III	3.20	2.90	3.10	3.00	3.05 ± 0.13
28	Toe IV length	TL IV	4.25	4.00	4.10	4.30	4.16 ± 0.14
29	Toe V length	TL V	3.05	2.95	3.15	3.05	3.05 ± 0.08
30	Toe I disk width	TD I	0.30	0.20	0.30	0.25	0.26 ± 0.05
31	Toe II disk width	TD II	0.35	0.25	0.35	0.30	0.31 ± 0.05
32	Toe III disk width	TD III	0.50	0.40	0.45	0.50	0.46 ± 0.05
33	Toe IV disk width	TD IV	0.65	0.56	0.60	0.60	0.60 ± 0.04
34	Toe V disk width	TD V	0.60	0.45	0.50	0.50	0.51 ± 0.06

Forelimb length shorter than hand length (FLL = 4.70; HAL = 4.90). Relative lengths of fingers I < II < IV < III (FL I = 1.20; FL II = 1.75; FL III = 3.40; FL IV = 2.15). Fingertips with well-developed discs (FD I = 0.25, FD II = 0.45, FD III = 1.1, FD IV = 1.2) bearing circum-marginal grooves. Dermal fringe absent on fingers. Webbing between fingers absent. Subarticular tubercles weak, number of subarticular tubercles in fingers: I = 1, II = 1, III = 1, IV = 1, rounded. Supernumerary tubercles indistinct. Nuptial pad absent.

Hind limbs long, shank shorter than thigh (ShL = 10.03; TL = 10.10), longer than foot (FOL = 7.95). Relative toe length I < II < V < III < IV (ToL I = 1.15; ToL II = 2.10, ToL III = 3.20; ToL IV = 4.25; ToL V = 3.05). Toes with well-developed discs (TD I = 0.30, TD II = 0.35, TD III = 0.50, TD IV = 0.65, TD V = 0.60). Webbing moderate, webbing formula (fingers: I2-2^+^II1¾-2^+^III1½-3IV2¾-2^−^V) (Fig. 5D, E). Inner and outer metatarsal tubercles absent, subarticular tubercle present (toe: I = 1, II = 1, III = 2, IV = 3, V = 2). Supernumerary tubercles absent.

In preservative, dorsum dark gray; loreal and tympanic regions lighter; forelimbs and hind limbs with black bands. Venter uniform cream white, vocal sac with dark gray flecks. Webbing cream; ventral side of feet and hands light gray with small black spots.

In life, dorsum grayish brown with dark brown specks; “)-(“ or “)(“ shaped blackish mark present on the mid dorsum; blackish line between upper eyelids; snout much darker, loreal and tympanic region blackish; iris dark golden brown. Dorsal side of hind limbs with several black bands; forelimbs with single band these bands are also present in the other members of this genus. Fingers and toes discs reddish or whitish. Abdomen brownish, with few black spots. Vocal sac translucent whitish, with a few black flecks. A few dark spots present near fore limbs. Foot webbing grayish.

##### Variation.

Because all specimens were males, sexual dimorphism could not be determined. Details of morphometric variation observed in four individuals are provided in Table 3. All of the specimens are almost similar except the size and the coloration. One of the four specimens (JnUZool-A0619) is smaller than others. For two specimens (JnUZool-A0619, JnUZool- A0519) the ventral dark gray flecks are more than others. The) (shape is present on the dorsum of three specimens where for one specimen (JnUZool- A0519) it is shaped “)-(“. Some individuals have a greater proportion of dark gray spots on the ventral surface. Detailed comparisons between *R. rezakhani* sp. nov. and other species of *Raorchestes* are provided below.

##### Bioacoustics analyses.

An advertisement call of the paratype (JnUZool-A0519) from the Lawachara National Park were recorded at an ambient air temperature of 27.8 °C, 97% relative humidity. Advertisement calls occurred without call groups (Fig. 3). The duration of the analyzed call was 16 s. The number of notes within this call was 25, and number of pulses within a note varied from 5–11 (8.84 ± 1.70 *SD*). Note duration was 0.183 – 0.379 s. The interval between notes was 0.222 – 0.592 s (0.323 ± 0.098 *SD*, *N* = 24). These intervals increase gradually within a call (mean interval for first five notes = 0.2422, mid five notes = 0.2784, last five notes = 0.4754). Pulse duration was 0.003–0.029 s (0.013 ± 0.007 *SD*, *N* = 205 pulses), duration of intervals between pulses was 0.005–0.127 s (0.027 ± 0.017 *SD*, *N* = 179 intervals). Pulse rate was 10–19/s (14.27 ± 2.49 *SD*, *N* = 15 seconds interval). The advertisement call had a dominant frequency at 4.32–4.77 kHz (4.55 ± 0.12 *SD*, *N* = 25). To the human ear, the calls sounded similar to cricket calls.

##### Distribution and natural history.

*Raorchestes rezakhani* sp. nov. was recorded from the semi-evergreen forests of northeastern Bangladesh. They were active with the onset of the rainy season in the month of April. We did not hear calls of this species after August. Frogs were found inside the primary and secondary forest mainly on the edge of streams and near man-made trails. They often use the hilly slopes during calling. Individuals perch on leaves and branches of small trees and on bamboo trunks (with diameters of 1.5–4 cm). Vocalizing individuals were perched 1–1.5 m above the forest floor. We usually heard the calls immediately after the sunset (ca. 1815 h in April) although calling activity started a little earlier when it was raining.

##### Comparisons.

Based on morphology, we compared *Raorchestes rezakhani* sp. nov. with some other member of this genus. This new species is differs from *R. amboli* (Biju & Bossuyt, 2009), *R. anili* (Biju & Bossuyt, 2006), *R. charius* (Rao, 1937), *R. chlorosomma* (Biju & Bossuyt, 2009), *R. flaviventris* (Boulenger, 1882), *R. glandulosus* (Jerdon, 1853), *R. jayarami* (Biju & Bossuyt, 2009), *R. kaikatti* (Biju & Bossuyt, 2009), *R. luteolus* (Kuramoto & Joshy, 2003), *R. munnarensis* (Biju & Bossuyt, 2009), *R. nerostagona* (Biju & Bossuyt, 2005), *R. ochlandrae* (Gururaj et al., 2007), *R. ponmudi* (Biju & Bossuyt, 2005), *R. signatus* (Boulenger, 1882), *R. sushili* (Biju & Bossuyt, 2009), *R. wynaadensis* (Jerdon, 1853), *R. kakachi* Seshadri et al., 2012, *R. crustai* Zachariah et al., 2011, *R. johnceei* Zachariah et al., 2011, *R. theuerkaufi* Zachariah et al., 2011, *R. thodai* Zachariah et al., 2011, *R. gryllus* (Smith 1924) by its smaller size. SVL of male individuals of these species ranged from 24.9–36.8 mm whereas *Raorchestes rezakhani* sp. nov. is 20.06 mm. *Raorchestes rezakhani* sp. nov. is quite similar to *R. longchuanensis*Yang et al. 1979 but differs for the following characters: tympanum indistinct in males (vs. distinct); snout sub-elliptical (vs. pointed); thigh shorter than the tibia/shank, TL/ShL = 93% (vs. Thigh slightly longer than the tibia/shank, TL/ShL = 97%); inner metatarsal tubercles absent (vs. present) [Yang et al. 1979; Al-Razi et al. 2020]. *Raorchestes rezakhani* sp. nov. differs from *R. tuberohumerus* in: snout sub-elliptical (vs. slightly pointed); relative lengths of fingers I < II < IV < III (vs. I < IV < II < III); thigh shorter than the tibia/shank, TL/ShL = 93% (vs. thigh longer than the tibia/shank, ShL/TL = 96%); inner metatarsal tubercles absent (vs. present); supernumerary tubercles feebly distinct (vs. distinct) [Kuramoto and Joshy 2003; Padhye et al. 2015]. *Raorchestes rezakhani* sp. nov. differs from *R. gryllus* in: snout sub-elliptical (vs. pointed); tympanum indistinct in males (vs. large and rounded); relative toe lengths I < II < V < III < IV (vs. I < II < III < V < IV); subarticular tubercles in finger weakly distinct I = 1, II = 1, III = 1, IV = 1 (vs. distinct I = 1, II = 1, III = 2, IV = 1) [Smith 1924; Orlov et al. 2012]. *Raorchestes rezakhani* sp. nov. is also similar to *R. shillongensis* (Pillai & Chanda, 1973) but differs in: SVL of male 20.06 ± 0.87 (vs. 16.51 ± 1.29); head wider than long, HL/HW = 61% (vs. length slightly greater than the width, HW/HL = 98%); snout length shorter than the eye diameter (vs. slightly longer than eye diameter); subarticular tubercles in finger weakly distinct, I = 1, II = 1, III = 1, IV = 1 (vs. distinct, I = 1, II = 1, III = 2, IV = 1) [Pillai and Chanda 1973; Boruah et al. 2018]. *Raorchestes rezakhani* sp. nov. is very similar to *R. parvulus* but differs in: forearm and hand length (9.05–9.95 mm) generally shorter than half body size (vs. longer than the half body size); relative toe length I < II < V < III < IV (vs. I < II < III < V < IV); toe subarticular tubercle: I = 1, II = 1, III = 2, IV = 3, V = 2 (vs. I = 1, II = 1, III = 2, IV = 2, V = 1); inner metatarsal tubercles absent (vs. present) [Boulenger 1893; Yu et al. 2019]. *Raorchestes rezakhani* sp. nov. differs from *R. sahai* (Sarkar & Ray, 2006) in: smaller SVL (18.85–20.90 vs. 25–26 mm); nostril closer to tip of snout than to eye, NS/EN = 67% (vs. equidistance from the tip of the snout and the eye NS/EN = 100%); snout length shorter than the eye diameter, SL/ED = 87% (vs. slightly longer than eye diameter, ED/SL = 81%); interorbital distance larger than the upper eyelid UEW/IOD= 65% (vs. equal to the upper eyelid, UEW/IOD = 100%) [Sarkar and Ray 2006]. *Raorchestes rezakhani* sp. nov. differs from *R. annandalii* in: snout sub-elliptical (vs. pointed); nostril closer to tip of snout than to eye, NS/EN = 67% (vs. equidistant from the tip of the snout and the eye, NS/EN = 100%); inner metatarsal tubercles absent (vs. feebly distinct); ShL longer than TL, TL/ShL = 93% (vs. ShL shorter than TL) [Boulenger 1906; Chanda 1994]. *Raorchestes rezakhani* sp. nov. differs from *R. menglaensis* (Kou 1990) in: male with external single subgular vocal sac (vs. internal single subgular vocal sac); outer metatarsal tubercle absent (vs. present); [Padhye et al. 2013; Kou 1990]. This new species differs from *R. garo* (Boulenger 1919) in: SVL 18.85–20.90 (vs. 13–16 mm); eye diameter larger than the interorbital distance, IOD/ED = 88% (vs. less than interorbital distance, ED/IOD = 92%); dark line present between eyelids (vs. absent); nostril closer to tip of snout than to eye, NS/EN = 67% (vs. equidistance from the tip of the snout and the eye or slightly closer to the tip of snout); tympanum indistinct (vs. distinct); inner metatarsal tubercles absent (vs. present) [Boulenger 1919; Chanda 2002]. *Raorchestes rezakhani* sp. nov. differs from *R. kempiae* (Boulenger 1919) in: SVL 18.85–20.90 (vs. 13–17.5 mm); nostril closer to tip of snout than to eye (vs. equidistant from the tip of the snout and the eye); tympanic fold indistinct (vs. distinct) [Boulenger 1919, Chanda 1994, 2002].

**Figure 6.**
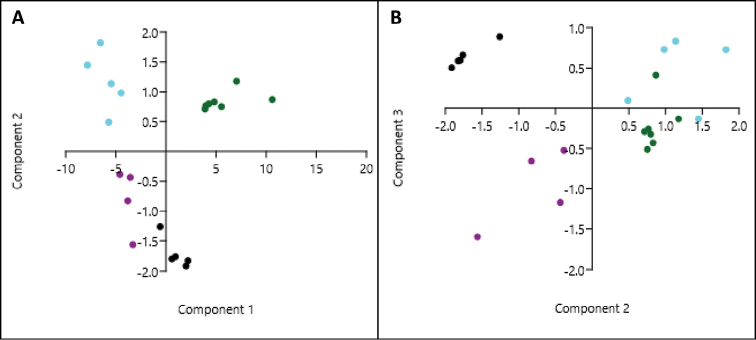
**A** Scatterplot of principle component axes 1 and 2 and **B** principle component axes 2 and 3. *R. tuberohumerus* (light blue), *R. gryllus* (green), *R. longchuanensis* (black), and *R. rezakhani* sp. nov. (purple).

Principle Components Analysis showed that the specimens of *R. rezakhani* sp. nov. did not overlap with *R. longchuanensis*, *R. tuberohumerus*, or *R. gryllus* (Fig. 6). Eigenvalues indicated that PC1 accounted for more than 91% of the variation in the data while PC2 contributed another 5% (Table 4). Thus, the inclusion of further principle components would not add substantially to the characterization of these species based on these variables. Loading of individual morphological variables indicated that SVL, HL, HW, THL and TL strongly influenced PC1, ED, SL, UEW and THL strongly influenced PC2, while HL and TL strongly influenced PC3, that helped to segregate the *R. rezakhani* sp. nov. from the remaining three species (Table 5).

**Figure 7.**
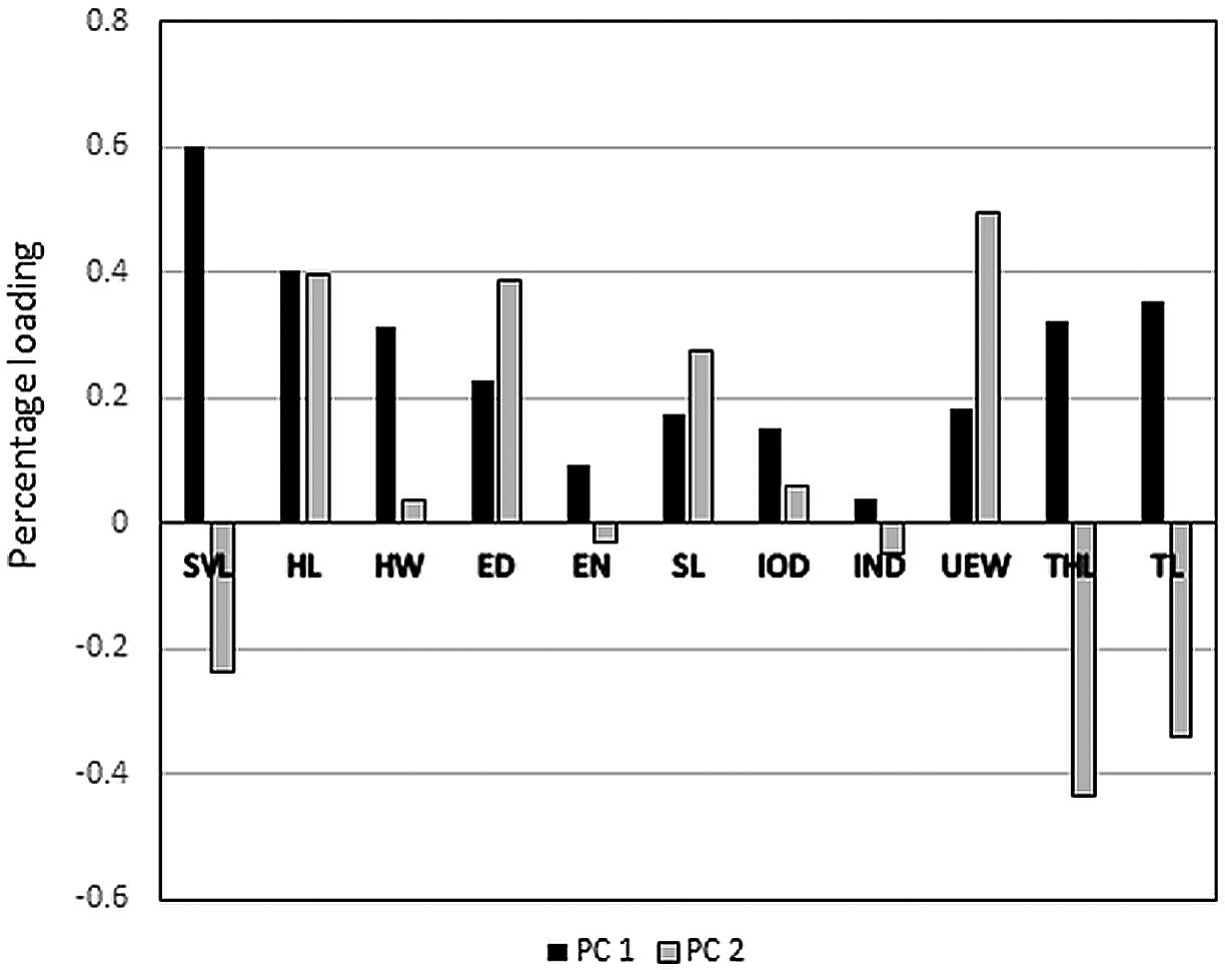
Plot showing individual loadings of each morphometric variable in relation to PC1, which accounted for over 91% of the variation in the data.

**Table 4.** Eigen analysis showing relative contributions of each Principle Component towards the characterization of each species.

Principle component	Eigen value	% variance
1	26.07	91.15
2	1.57	5.50
3	0.47	1.66
4	0.28	0.98
5	0.07	0.25
6	0.06	0.21
7	0.04	0.15
8	0.01	0.04
9	0.0064	0.02
10	0.0043	0.01
11	0.0019	0.006

**Table 5.** Loading plot showing individual loadings of each measured variable in *Raorchestes tuberohumerus*, *R. gryllus*, *R. longchuanensis*, and *R. rezakhani* sp. nov. against four principle components.

Variable	PC 1	PC 2	PC 3	PC 4
SVL	0.60	-0.24	-0.25	-0.69
HL	0.40	0.40	0.47	-0.07
HW	0.31	0.04	0.12	0.10
ED	0.23	0.39	-0.36	0.21
EN	0.09	-0.03	0.23	0.03
SL	0.18	0.27	0.11	0.19
IOD	0.15	0.06	0.24	0.01
IND	0.04	-0.05	0.32	0.00
UEW	0.18	0.50	-0.26	0.14
THL	0.32	-0.43	0.33	0.41
TL	0.35	-0.34	-0.41	0.49

## Discussion

Our discovery of a new species of *Raorchestes* is not unexpected (Reza 2014; Khan 2015; IUCN 2015). Our recent report of *R. longchuanensis* from northeastern Bangladesh (Al-Razi et al. 2020) supports the suggestion of the authors of the species, who stated that it was very likely to occur outside of Longchuan (the type locality) as well as nearby provinces in southern China (Yang et al. 2004). We suggested that the broad similarities between southern China, northern Myanmar, several northeastern states of India, and northeastern Bangladesh with their relative proximity to each other would suggest that many species may occur across this region (Al-Razi et al. 2020). Slik et al. (2018) recently classified the world’s forest types using phylogenetic similarities into five floristic regions. Two of these five regions, namely the Indo-Pacific and the Subtropical floristic regions, are of interest. The Indo-Pacific region spans across the Indian subcontinent and through Myanmar into the rest of Southeast Asia. In addition, the Subtropical floristic region spans from northeastern India, northern Myanmar, through southern China (where it has significant overlaps with the Indo-Pacific floristic region) further into eastern China (Slik et al. 2018). The Indian subcontinental fauna differs considerably compared to the Southeast Asian fauna, making the entire region of great interest to diversification of biota.

The Western Ghats region of India is a global biodiversity hotspot (Gadgil 1996). The region has undergone biodiversity loss along with changes in land use that has contributed towards the creation of geographic barriers within the last few decades (Gadgil 1996). The diversification of frogs in the Western Ghats has generally been attributed to long-term ecological change over extended geological time scales (Priti et al. 2016). Due to its status as a biodiversity hotspot, considerable research attention has been placed on this region, resulting in more discoveries in the anuran fauna. On the other hand, the taxonomic challenges as well as the lack of funding for dedicated studies examining species diversity in Bangladesh could have precluded the detection of cryptic species until recently (Reza 2014; Khan 2015; IUCN 2015; Frost 2020). This is also true for the northeastern regions of India, where relatively few studies have been done on cryptic anurans (Ao et al. 2003; Vijayakumar et al. 2016; Boruah et al. 2018). Myanmar has only recently been opened up to biological exploration and we anticipate that more species will be found from this region. Renewed interest, especially with respect to anuran biodiversity and the relative availability and cost-effectiveness of molecular tools, have made it easier to target cryptic species for identification. We anticipate that further extensive surveys followed by molecular characterization, and bioacoustics data could aid in discovering additional species and delineating their occurrence in the region (Vijaykumar et al. 2014; Priti et al. 2016).

Northeastern India, particularly Meghalaya and parts of Assam, are separated by the river Brahmaputra, that effectively creates differences in forest type (Champion and Seth 1968). Areas south of the river have more subtropical influence, compared to areas north of the river, which are climatically affected by the Himalayas and its foothills, due to variation in local climatic patterns (Champion and Seth 1968). Thus, there are forested areas in Meghalaya, Assam, Tripura and Mizoram states of India and Bangladesh with variation in niche types affected by local climates that could have encouraged diversification of *Raorchestes* or other forest-dwelling genera (Ahmed et al. 2009; Vijayakumar et al. 2016).

The similarities between *R. rezakhani* sp. nov., *R. tuberohumerus*, and *R. gryllus* could offer some insight into the diversification of the *Raorchestes* in the region. *Raorchestes tuberohumerus* is distributed in the Western Ghats while *R. gryllus* is limited in distribution to central Vietnam and Laos (Frost 2020). *Raorchestes shillongensis*, restricted to a small part of Megalaya state of India, is mostly closely related to either *R. tuberohumerus* (*p* = 3.7%) and *R. indigo* (*p* = 3.9%), a species also found in the Western Ghats (Frost 2020). Thus, we suggest that the *Raorchestes* species in northeastern India and surrounding regions may have separated from Western Ghats species giving rise to *R. shillongensis* and *R. rezakhani* sp. nov. relatively recently (Vijayakumar et al. 2016). Ancestors of *Raorchestes parvulus* may have diverged from Western Ghats stock even earlier (difference compared to *R. bombayensis* was 4.4%) and from Eastern Ghats and Deccan plateau species (difference compared to *R. sanctisilvaticus* was 4.2%). Our analysis indicates that *R. longchuanensis* is quite distinct from *R. parvulus* (*p* = 6.5%). *Raorchestes rezakhani* sp. nov. is also significantly different both morphologically and genetically from *R. parvulus*, providing support of the idea that *R. parvulus* is part of a Southeast Asian species complex. The status of *Raorchestes annandalii* is not clear since there are no sequences of 16S rRNA genes for this species in GenBank. They are morphologically distinct from *R. rezakhani* sp. nov. and we speculate that they could be part of a species complex associated with northeast India and northern Myanmar, and may include species such as *R. shillongensis*, *R. longchuanensis*, and *R. rezakhani* sp. nov. as closely related congenerics. Further genetic analyses could clarify their status in relation to the evolution and biogeography of *Raorchestes* in the region. We speculate that *R. rezakhani* sp. nov. may be found in other adjoining areas including the northeastern states of India and northern Myanmar due to close affiliations of the habitat types in this floristic region.

It is also important to note that Bangladesh retains some forest patches that are of high value to biodiversity. The two areas in the northeast, Lawachara National Park and Adampur reserve forest contain high bird and mammal diversity. Six of the ten species of primates in Bangladesh occur there in numbers higher than elsewhere in the country (Al-Razi et al. 2019; Al-Razi and Maria 2019). Lawachara is legally protected whereas Adampur is under the management of the Forest Department of the Ministry of Environment and Forests of Bangladesh, but not under formal protected areas status. Illegal logging, fuel wood collection, and hunting occurs in these areas (Muzaffar et al. 2011; Islam et al. 2013). Although a signatory to the Convention on Biological Diversity, all forested and other wilderness areas suffer from poor implementation of the principles of ecosystem management (Muzaffar et al. 2011). Lawachara has a total area of about 12 km^2^ and Adampur has an area of about 71.9 km^2^, making both of them relatively small patches. Despite all odds, our finding of new species and previous studies on primates suggest that viable populations of varied species persist in these areas (Muzaffar et al. 2007, 2011; Al-Razi et al. 2020). Thus, efforts must be made to protect these remaining forest patches, which may still retain undiscovered new species, as documented in this study.

## Ethics statement

Fieldwork and sampling were carried out in Adampur Reserve Forest and Lawachara National Park, with permission from Forest Department Bangladesh (Permit no. 22.01.0000.101.23.2019.2940). Individuals were euthanized and muscle tissue was collected in strict accordance with protocols approved by the Forest Department solely for scientific research. The sampling is unlikely to affect population size of the species since the bare minimum of specimens were collected.

## Supplementary Material

XML Treatment for Raorchestes rezakhani
